# Are intravitreal injections essential during the COVID-19 pandemic? Global preferred practice patterns and practical recommendations

**DOI:** 10.1186/s40942-022-00380-6

**Published:** 2022-06-07

**Authors:** A. C. S. Tan, R. Schwartz, D. Anaya, I. Chatziralli, M. Yuan, M. V. Cicinelli, L. Faes, M. Mustapha, N. Phasukkijwatana, D. Pohlmann, R. Reynolds, A. Rosenblatt, A. Savastano, S. Touhami, K. Vaezi, C. V. Ventura, D. Vogt, J. Ambati, M. D. de Smet, A. Loewenstein, the International Retina Collaborative

**Affiliations:** 1 Singapore National Eye Centre, Singapore, Singapore; 2 Singapore Eye Research Institute, Singapore, Singapore; 3 Duke-NUS Medical School, National University of Singapore, Singapore, Singapore; 4 Moorfields Eye Hospital NHS Foundation Trust, London, UK; 5 Department of Retina, Clínica de Oftalmología de Cali, Valle del Cauca, Colombia; 6 2nd Department of Ophthalmology, National and Kapodistrian University of Athens, Athens, Greece; 7 Department of Retina, Zhongshan Ophthalmic Center, Sun Yat-Sen University, Guangzhou, China; 8 School of Medicine, Vita-Salute San Raffaele University, Milan, Italy; 9 Department of Ophthalmology, IRCCS San Raffaele Scientific Institute, Milan, Italy; 10 Cantonal Hospital Lucerne, Lucerne, Switzerland; 11 Department of Ophthalmology, Universiti Kebangsaan Malaysia, Kulala Lumpur, Malaysia; 12 Department of Ophthalmology, Faculty of Medicine Siriraj Hospital, Mahidol University, Bangkok, Thailand; 13 Charité – Universitätsmedizin Berlin, FreieUiversität Berlin, Humboldt-Universität zu Berlin, and Berlin Institute of Health, Berlin, Germany; 14 Department of Ophthalmology, Aneurin Bevan University Health Board, Wales, UK; 15 Department of Ophthalmology, Tel-Aviv Sourasky Medical Center Tel-Aviv, Israel Sackler Faculty of Medicine, Tel-Aviv University, Tel-Aviv, Israel; 16 Ophthalmology Department, Fondazione Policlinico Universitario A. Gemelli IRCCS, Rome, Italy; 17 Università Cattolica del Sacro Cuore, Rome, Italy; 18 Department of Ophthalmology, Reference Center in Rare diseases, DHU Sight Restore, Hôpital Pitié Salpêtrière, Sorbonne Université, 47-83 Boulevard de l’Hôpital, 75013 Paris, France; 19 Department of Ophthalmology and Visual Sciences, University of British Columbia, Vancouver, Canada; 20 Department of Ophthalmology, Altino Ventura Foundation (FAV), Recife, Brazil; 21 Department of Ophthalmology, HOPE Eye Hospital, Recife, Brazil; 22 Department of Ophthalmology, Ludwig-Maximilians-University, Munich, Germany; 23 Center for Advanced Vision Science, Department of Ophthalmology, University of Virginia School of Medicine, Charlottesville, USA; 24 Department of Ophthalmology, Leiden University, Leiden, The Netherlands; 25 MIOS sa, Lausanne, Switzerland

**Keywords:** COVID-19, Intravitreal injections, Age-related macular degeneration, Diabetic macula edema, Practice patterns, Recommendations

## Abstract

Tertiary outpatient ophthalmology clinics are high-risk environments for COVID-19 transmission, especially retina clinics, where regular follow-up is needed for elderly patients with multiple comorbidities. Intravitreal injection therapy (IVT) for chronic macular diseases, is one of the most common procedures performed, associated with a significant burden of care because of the vigorous treatment regimen associated with multiple investigations. While minimizing the risk of COVID-19 infection transmission is a priority, this must be balanced against the continued provision of sight-saving ophthalmic care to patients at risk of permanent vision loss. This review aims to give evidence-based guidelines on managing IVT during the COVID-19 pandemic in common macular diseases such as age-related macular degeneration, diabetic macula edema and retinal vascular disease and to report on how the COVID-19 pandemic has affected IVT practices worldwide.

To illustrate some real-world examples, 18 participants in the International Retina Collaborative, from 15 countries and across four continents, were surveyed regarding pre- and during- COVID-19 pandemic IVT practices in tertiary ophthalmic centers. The majority of centers reported a reduction in the number of appointments to reduce the risk of the spread of COVID-19 with varying changes to their IVT regimen to treat various macula diseases. Due to the constantly evolving nature of the COVID-19 pandemic, and the uncertainty about the normal resumption of health services, we suggest that new solutions for eye healthcare provision, like telemedicine, may be adopted in the future when we consider new long-term adaptations required to cope with the COVID-19 pandemic.

## Background

As the novel severe acute respiratory syndrome coronavirus 2 (SARS-CoV-2)-induced COVID-19 [1] emerged as a global pandemic with significant morbidity and mortality, massive disruptions in healthcare, financial, and social sectors have occurred [1]. To allow healthcare systems to adequately cope with COVID-19, governments around the world have placed strict measures in place to curb the spread of the disease.

Ophthalmologists are at particularly high risk due to their close proximity to patients during slit-lamp and indirect ophthalmoscope evaluations [2]. There is also a risk of virus transmission through aerosol contact with the conjunctiva and exposed mucous membranes [2]. Retinal providers and clinics face additional challenges in crowded clinics with predominantly elderly patients, who have multiple comorbidities, requiring multiple investigations and long-waiting times [3]. Furthermore, most of these patients have sight-blinding chronic diseases such as neovascular age-related macular degeneration (nAMD), diabetic macula edema (DME), and macular edema associated with retinal vascular occlusion (ME-RVO), necessitating frequent intravitreal injection therapy (IVT), imposing a substantial burden on physicians, staff, patients, and caregivers, even in routine care.

The COVID-19 pandemic imposes additional barriers to the management of retinal diseases, in terms of non-adherence to long-term treatment and follow-up regimens [4]. In addition, many health authorities and hospital management teams have mandated that, during this high-risk COVID-19 period only urgent and emergent care should be provided and that all routine clinical activity be deferred, to allow redirection of available resources to those at high risk for permanent visual loss [5, 6].

This study aims to summarize the literature, current guidelines, and evidence-based recommendations with regards to managing IVT during the COVID-19 pandemic and report on the effect of the COVID-19 pandemic on the visual outcomes, number of injections and adherence to follow up in IVT patients. In addition, we illustrate variability in changes to IVT practices in response to the COVID-19 pandemic in the early days from examples of tertiary ophthalmic centers worldwide and provide updated evidence on recommended best practices for IVT regimens and administration.

## Methods

A comprehensive literature review was performed based on a search of previous published papers (including original articles, reviews, editorials) in English, relevant to medical retina management or IVT treatment during the COVID-19 pandemic (keywords: guidelines, COVID-19, SARS-CoV-2, intravitreal injections, medical retina, age-related macular degeneration, diabetic macula edema, retinal vein occlusion) up to 22nd April 2022, available on the PubMed database and included published guidelines from various professional ophthalmology societies (e.g. American Academy of Ophthalmology, Royal College of Ophthalmologists, United Kingdom, Canadian Retinal Society). Data were stored using Microsoft Excel (Microsoft, Redmond, WA), and absolute and relative (%) numbers are presented.

To illustrate real-world examples of the varied effect of the COVID-19 pandemic on routine IVT practice, 18 participants in the International Retina Collaborative, from 18 different cities in 15 countries and across four continents, were surveyed regarding pre- and during- COVID-19 pandemic IVT practices in tertiary ophthalmic centers (Tables 1, 2, 3). The responses were collected from 24^th^ March to 22nd April 2020 (last response update). Participant agreement/consent was implied by completion or return of the questionnaire.

**Table 1:** Summary of the global routine intravitreal injection therapy (IVT) practices during the pre-COVID-19 pandemic time

Country (city/region)	Type of institution	Prior to intravitreal injections being administered			Intravitreal Injection procedure			
		Imaging with OCT done at every visit		Ophthalmology consult performed at every visit	Setting where the majority of IVTs are performed	Skilled manpower used to administer IVT	Routine equipment used to administer IVT (Surgical mask, drape, gown, sterile gloves, speculum, iodine)	Bilateral injections allowed on the same day
Asia and Pacific Region								
China (Guangzhou)	Tertiary stand-alone ophthalmology centre		Yes	Yes	Operating theatre	Senior/Junior Ophthalmologists	All	No
Israel (Tel Aviv)	Ophthalmology department within General Hospital		No^a^	No^a^	Separate treatment room	Senior/Junior Ophthalmologists	All except gowns	Yes
Malaysia (Kuala Lumpur)	Ophthalmology department within General Hospital		Yes	Yes	Separate treatment room	Junior ophthalmologists	All	Yes
Singapore	Tertiary stand-alone ophthalmology centre		No^a^	No^a^	Separate treatment room	Senior/Junior Ophthalmologist, Specialised nurses	All except gowns	Yes
Thailand (Bangkok)	Ophthalmology department within General Hospital		No^a^	No^a^	Separate treatment room or stand-alone IVT clinics	Senior/Junior Ophthalmologists/Residents	All except gowns	Yes
Europe								
France (Paris)	Ophthalmology department within General Hospital		Yes	Yes	Separate treatment room	Junior ophthalmologist	All	Yes
Germany (Berlin)	Tertiary stand-alone ophthalmology centre		No^a^	No^a^	Operating theatre	Senior ophthalmologist	All except gowns	No
Germany (Munich)	Tertiary stand-alone ophthalmology centre		Yes	Yes	Separate treatment room/ Operating theatre	Junior/Senior ophthalmologist	All except gowns	Yes
Greece (Athens)	Ophthalmology department part of General Hospital		Yes	Yes	Separate treatment room	Senior/Junior Ophthalmologists	All except gowns	Yes
Italy (Rome)	Ophthalmology department part of General Hospital		No (only after 3 loading doses)	No (only after 3 loading doses)	Separate treatment room / Operating theatre	Senior ophthalmologist	All except gown (drape recommended)	No
Italy(Milan)	Ophthalmology department part of General Hospital		No^a^	No^a^	Separate treatment room/Operating theatre	Senior/Junior Ophthalmologists	All	No
Switzerland (Lucerne)	Ophthalmology department part of General Hospital		No	No (only at fixed time points)	Operating theatre	Junior Ophthalmologist, Specialised nurses	All	Yes
United Kingdom(London)	Tertiary stand-alone ophthalmology centre		Yes	Yes	Separate treatment room	Senior/Junior Ophthalmologist, Specialised nurses	All	Yes
United Kingdom (Wales)	Ophthalmology department part of General Hospital		Yes	Yes (most done virtually)	Within the outpatient clinic	Senior/Junior Ophthalmologist, Specialised nurses	All (drape and masks only recommended)	Yes
North and South America								
Brazil (Recife)	Tertiary stand-alone ophthalmology centre		Yes	Yes	Operating theatre	Senior/Junior Ophthalmologist	All	Yes
Canada (Vancouver)	Tertiary stand-alone ophthalmology centre		Yes	Yes	Within the outpatient clinic	Senior Ophthalmologist	Iodine only (some substitute chlorhexidine for iodine), speculum optional	No
Colombia (Cali)	Tertiary stand-alone ophthalmology centre		Yes	Yes	Within the outpatient clinic	Senior/Junior Ophthalmologist	All	Yes
United States of America (Chicago)	Ophthalmology department part of General Hospital		Yes	Yes	Within the outpatient clinic	Senior ophthalmologist	All except gown	Yes (rarely)

^a^IVT also administered in injection only clinics/appointments with no imaging or ophthalmologist consult

## Results

### Global pre-pandemic routine IVT practice and the effects of the COVID-19 pandemic on the IVT practices surveyed.

**Fig. 1:**
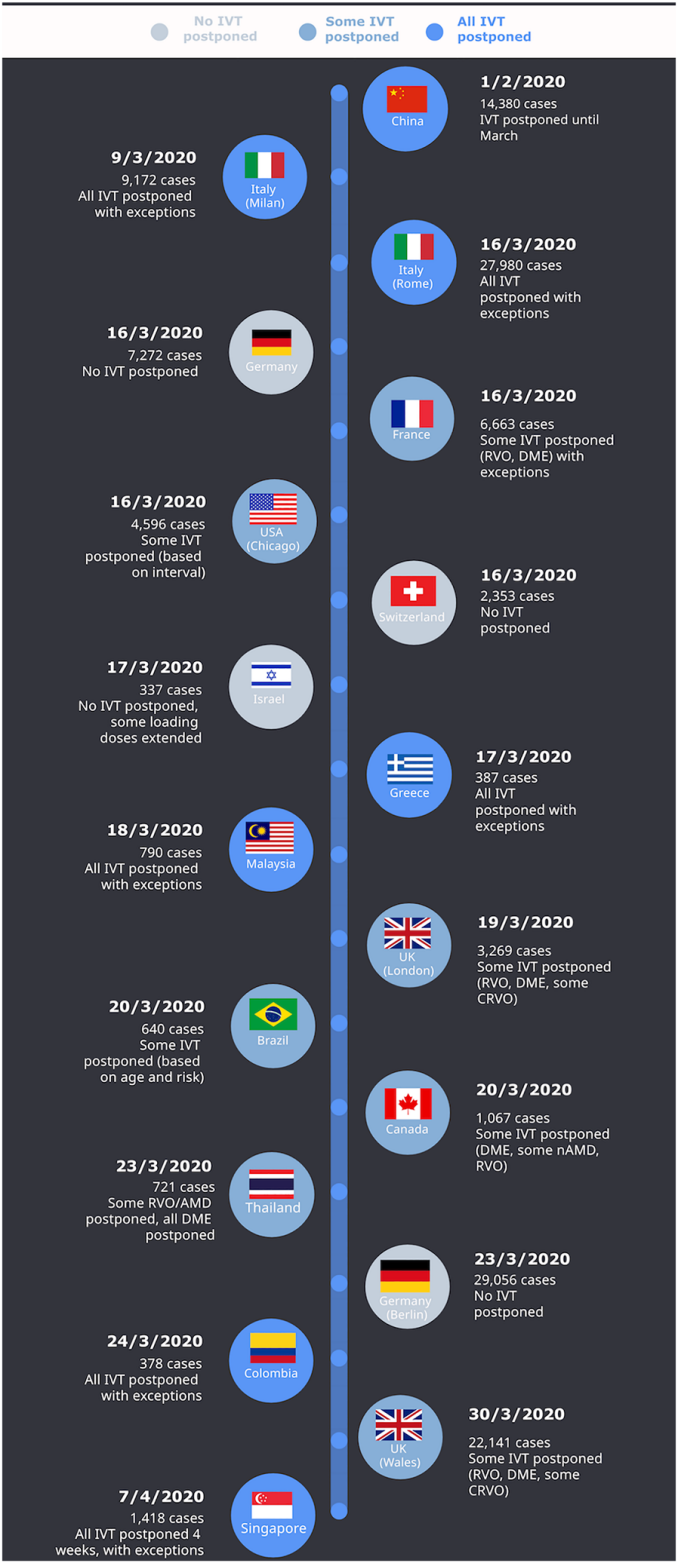
Timeline of the dates when changes to IVT practice occurred in the different centers surveyed and the number of COVID-19 cases on that date

Routine clinical examination, investigations and IVT procedures performed pre-pandemic were summarized in Table 1. The approximate dates in which changes to the IVT practices occurred in respective countries are summarized in Table 2 and Fig. 1. The changes to the appointments with regards to diagnosis (nAMD, DME and ME-RVO), changes to clinical assessments and PPE use in various centers is summarized in Table 3. Factors that were likely to influence the decision to implement changes in IVT practice included: the date when the first case of COVID-19 was detected, the rate of COVID-19 infection in the community, the speed of response of the respective governments in implementing lockdown policies, and the availability of the healthcare resources (Table 2). The most common reasons cited for implementing these changes included the high risk of COVID-19 transmission and the need to comply with hospital policies. Less common reasons included a lack of manpower and resources.

**Table 2:** The global timeline of when changes to intravitreal injection therapy (IVT) practices were instituted during the COVID-19 pandemic, in the context of the magnitude of the COVID-19 problem in various countries

Country (city)	Estimated date the changes started	Number of cases of COVID-19 in the country on that date^a^	Other restrictions within the country at that date	Main reasons for the change in practice	Changes in practice patterns during the COVID-19 pandemic with regards to various chronic macula diseases receiving IVT		
					nAMD	DME	ME-RVO
*Asia and Pacific Region*							
China (Guangzhou)	1/2/2020	14,380	Travel ban, lockdown	High risk of hospital transmitted infections	All IVT postponed in February, given in March	All IVT postponed in February, given in March	All IVT postponed in February, given in March
Israel (Tel Aviv)	17/3/2020	337	Travel ban, close borders, lockdown	High risk of hospital transmitted infections	No IVT injections postponed, some loading doses could be extended	No IVT injections postponed, some loading doses could be extended	No IVT injections postponed, some loading doses could be extended
Malaysia (Kuala Lumpur)	18/3/2020	790	Travel ban, lockdown	High risk of hospital transmitted infections	All IVT postponed with exceptions	All IVT postponed with exceptions	All IVT postponed with exceptions
Singapore	7/4/2020	1418	Travel ban, close borders, partial lockdown	High risk of hospital transmitted infections	All IVT postponed for 4 weeks except patients with only 1 seeing eye can receive IVT	All IVT postponed for 4 weeks exceptions based on clinician discretion	All IVT postponed for 4 weeks exceptions based on clinician discretion
Thailand (Bangkok)	23/3/2020	721	Travel ban, partial lockdown	High risk of hospital transmitted infection, Lack of resources	Some IVT postponed except those based on individual clinician’s discretion	All IVT postponed for 2–3 months	Some IVT injections postponed based on individual clinician’s discretion
*Europe*							
France (Paris)	16/3/2020	6663	Complete lockdown, travel ban	High risk of hospital transmitted infections	No IVT injections postponed	All postponed for 2/3 months except in single eye patients or threatening situations	All postponed for 2/3 months except in single eye patients or threatening situations
Germany (Berlin)	23/3/2020	29056	Travel ban Reduce close contacts, schools closed	High risk of hospital transmitted infection	No IVT injections postponed	No IVT injections postponed	No IVT injections postponed
Germany (Munich)	16/3/2020	7272	Travel ban, Reduce close contacts, schools closed	High risk of hospital transmitted infection	No IVT injections postponed	No IVT injections postponed	No IVT injections postponed
Greece (Athens)	17/3/2020	387	Travel ban, Lockdown, schools closed	High risk of hospital transmitted infection	All IVT injections postponed, exceptions allowed based on clinician’s discretion	All IVT injections postponed	All IVT injections postponed, exceptions allowed based on clinician’s discretion
Italy (Rome)	16/3/2020	27980	Travel ban, Lockdown, Close borders	Hospital policy, High risk of hospital transmitted infection	All IVT postponed except patients with only 1 seeing eye can receive IVT	All IVT postponed	All IVT postponed
Italy (Milan)	9/3/2020	9172	Travel ban, Lockdown, Close borders	High risk of hospital transmitted infection	All IVT postponed except patients with only 1 seeing eye can receive IVT	All IVT postponed	All IVT postponed except patients with neovascular glaucoma
Switzerland (Lucerne)	16/3/2020	2353	Travel ban, Lockdown, Close borders	High risk of hospital transmitted infection	No IVT injections postponed	No IVT injections postponed	No IVT injections postponed
United Kingdom (London)	19/3/2020	3269	Travel ban, Lockdown, Close borders	High risk of hospital transmitted infection	No IVT injections postponed but to continue on a fixed treatment regiment	All IVT postponed for 6 months	All IVT postponed for 6 months for BRVO, IVT given to CRVO based on clinician discretion
United Kingdom (Wales)	30/3/2020	22,141	Travel ban, Lockdown, Close borders	High risk of hospital transmitted infection	No injections postponed – Extended by 4 weeks rather than 2 where needed	No injections postponed – Extended by 4 weeks rather than 2 where needed	No injections postponed – Extended by 4 weeks rather than 2 where needed
*North and South America*							
Brazil (Recife)	20/3/2020	640	Travel ban, quarantine	High risk of hospital transmitted infectionFlatten the curve	IVT injections postponed in elderly and high-risk patients if vision and OCT were stable on last visit	Some IVT injections postponed in elderly and high-risk patients if vision and OCT were stable on last visit	Some IVT injections postponed in elderly and high-risk patients if vision and OCT were stable on last visit
Canada (Vancouver)	20/3/2020	1067	Travel Ban, State of Emergency, Close borders	High risk of hospital transmitted infection	Some IVT injections postponed for 3-month stable patients	All IVT injections postponed	Some IVT injections postponed for 3-month stable patients
Colombia (Cali)	24/3/2020	378	Travel Ban, State of Emergency, Lockdown, Close borders	Hospital policy, High risk of hospital transmitted infection	All IVT injections postponed for at least 1 month, exceptions allowed based on clinician’s discretion	All IVT injections postponed for at least 1 month, exceptions allowed based on clinician’s discretion	All IVT injections postponed for at least 1 month, exceptions allowed based on clinician’s discretion
United States of America (Chicago)	16/3/2020	4596	Travel restriction, lockdown	High risk of hospital transmitted infection Limited manpower	Some IVT postponed for patient with long IVT intervals, patients with shorter IVT maintained according to clinician’s discretion	Some IVT postponed for patient with long IVT intervals, patients with shorter IVT maintained according to clinician’s discretion	Some IVT postponed for patient with long IVT intervals, patients with shorter IVT maintained according to clinician’s discretion

nAMD: neovascular AMD, DME: Diabetic macula edema, ME-RVO: macula edema related to retinal vein occlusion, BRVO: branch retinal vein occlusion, CRVO: central retinal vein occlusion

^a^Data obtained from ref 73: https://www.worldometers.info/coronavirus/ unless specified otherwise

**Table 3:** Summary of changes to logistics and procedural practices of intravitreal injection therapy (IVT) during the COVID-19 pandemic time

Country (city/region)	Prior to intravitreal injections being administered				Intravitreal Injection procedure		
	Screening for high risk COVID patients performed (temperature screen, symptoms, travel history)	High-risk COVID patients allowed to the specialist outpatient clinic for IVT	Changes to performing OCT imaging	Changes to performing ophthalmology consult	Changes to the setting where the majority of IVTs was performed	Changes to the skilled manpower used to administer IVT	Changes to the IVT administration procedure or equipment
*Asia- Pacific*							
China(Guangzhou)	Yes	No	No	No	Yes, reduced numbers, social distancing, reduced follow up appointments	No	No
Israel(Tel Aviv)	Yes	No, deferred for 2 weeks	Yes, reduced OCT performed	Yes, reduced VA, slit-lamp exam	Yes, Reduced numbers and social distancingAn additional injection clinic opened outside the hospitalHome injections in selected cases	No	Yes, face shield and gown worn. N95 mask was available at physician’s discretion
Malaysia (Kuala Lumpur)	Yes	No, deferred	No	No	Yes, reduced numbers, social distancing, reduced follow up appointments	No	Yes, face shield was worn
Singapore	Yes	No, deferred	Yes, reduced OCT performed	Yes, reduced VA, slit-lamp exam	Yes, reduced numbers, reduced time in clinic, social distancing	No	Yes, goggles of face-shield recommended
Thailand (Bangkok)	Yes	Yes, if no fever detected	No	No	Yes, IVT clinic/OT stopped only IVT in the treatment room	No	No
*Europe*							
France(Paris)	Yes	Yes	Yes, reduced OCT performed (no OCT in patients with known interval)	Yes, no slit lamp exam in patients with known interval	Yes, reduced numbers, reduced time in clinic, social distancing	No	No
Germany (Berlin)	Not formally	Yes, obviously sick patients asked to return later	No	Yes, telephone consults for patients instead of routine follow up examination	Yes, reduced numbers	No	No
Germany(Munich)	Yes	Yes, high risk cases screened in isolation	No	Yes, only VA, IOP, OCT taken no slit lamp exam	Yes, reduced numbers	No	No
Greece(Athens)	Yes	No	Yes, reduced OCT performed	Yes, reduced VA and slit-lamp exam	Yes, reduced numbers, social distancing	No	No
Italy(Rome)	Yes	No, deferred for 2 weeks	No	No	Yes, reduced numbers, social distancing	No	Yes, face shields worn by all staff
Italy(Milan)	Yes	Yes, high risk cases screened in isolation	No	Yes, telephone consults for symptoms screening	Yes, no injections in OT all IVT done in small procedures room	Yes, more senior ophthalmologists performing IVT as junior staff are deployed elsewhere	Yes, face shields worn by all staff
Switzerland (Lucerne)	No	Yes	Yes, reduced OCT, done only in treatment naïve patients and those patients with significant vision loss	Yes, no routine VA, IOP and slit lamp examination telephone consults done	Yes, reduced numbers, waiting time, social distancing	No	No
United Kingdom(London)	Yes	No, deferred for 2 weeks	Yes, reduced OCT performed	Yes, no routine VA, IOP or slit lamp exam performed	Yes, reduced numbers, waiting time, social distancing	No	No
United Kingdom (Wales)	Yes (department dependent)	No, deferred for 2 weeks	Yes, reduced OCT performed	Yes, no routine VA, IOP or slit lamp exam performed, virtual consults continue	Yes, reduced numbers, waiting time, social distancing	No	Yes, surgical mask strongly recommended
*North and South America*							
Brazil(Recife)	Yes	No	No	Yes, included virtual consultations	Yes, reduced numbers, waiting time, social distancing	No	No
Canada(Vancouver)	No	Yes	No	Yes, DME and RVO patients contacted by telephone	No	No	Yes, gloves, goggles and masks for all staff, masks for any sick patients
Colombia(Cali)	Yes	No	Yes, reduced OCT performed	Yes, no pinhole or IOP, virtual consults where possible	No	No	Yes, face shield and gown worn. N95 mask was available at physician’s discretion
United States of America (Chicago)	Yes	Yes, high risk cases screened in isolation	Yes, only basic OCT allowed	No	Yes, reduced numbers, waiting time, social distancing	No	Yes

VA: visual acuity, IOP: intraocular pressure, OCT: optical coherence tomography, DME: diabetic macula edema, RVO: retinal vein occlusions

## Discussion

The multiple tertiary ophthalmic centers around the world included in our study reported varying responses to changes in their IVT practice in response to the COVID-19 pandemic, with the majority of centers reducing the number of appointments to reduce the risk of the spread of COVID-19 among staff and patients. Most centers reported having routine screening for high risk COVID-19 patients and about half of the tertiary centers reported the additional use of PPE for IVT procedures (in most of the other centers full PPE was already worn pre-COVID-19 pandemic).

### The recommended best practice guidelines for IVT during the COVID-19 pandemic.

The main guiding principles of planning IVT treatment in times of the COVID-19 pandemic include (1) minimizing the risk of COVID-19 infection between healthcare workers and patients; (2) continuing to provide IVT to patients to prevent permanent vision loss from the progression of their chronic macular disease [3, 7]. In addition, these decisions should be made in the context of other factors such as the number of COVID-19 cases within the country, the risk of COVID-19 transmission, the availability of healthcare resources, and government policies.

A recent paper by the Vision Academy Steering Committee outlined various guidelines on the treatment regimens for various common macular diseases requiring IVT, during the time when the COVID-19 pandemic began [5, 7]. External factors, such as the strain on the healthcare system caused by the pandemic, government-imposed restrictions, and the need to reduce the risk of virus transmission all led to global recommendations in IVT delivery: the number of visits should be kept to a minimum, the time within visits shortened, exposure should be minimized to the lowest number of the staff, and priority should be given to patients at greatest risk of vision loss [2, 7]. One strategy proposed in some of our surveyed centers and in previous studies is having two types of appointments: (1) an assessment appointment performed at baseline, after the 3rd injection of the anti-VEGF loading-dose, at regular intervals after, and at physician discretion in case of reported vision loss, consisting in a VA assessment, slit-lamp examination, and OCT and (2) an injection-only appointment, where IVT only is performed without any eye assessments [3, 7].

A proactive T&E regimen is ideal during the COVID-19 pandemic, as it reduces the number of visits and injections while maintaining visual outcomes [8]. However, a disadvantage of T&E is that the decision about the next treatment interval is made based on VA measurement and OCT findings, which need to be done at every visit, adding to the time spent in the clinic and increased close contact with patients [9]. Table 4 summarizes the benefits, risks, and recommendations for each routine assessment procedures done prior to IVT. Newer intravitreal drugs or drug delivery systems such as faricimab and port-delivery systems that are being currently developed aims to increased injection intervals with the potential to further reduce the number of clinic visits [10–12].

**Table 4:** Benefits, risks and recommendations for assessment procedures done prior to administering intravitreal injection treatment

Procedure	Benefits	Risks during COVID-19 pandemic	Risk of deferring procedure	Situations where the procedure is indicated	Situations where the procedure can be deferred	Modifications to the procedure during COVID-19 pandemic
VA testing	Widely accepted functional visual assessment Can be used to determine T&E decisions	Increasing contact time with patient and staff	Patients may not report vision loss Visual outcomes less closely monitored	Treatment naïve patients Patients who complain of visual loss	Patients receiving loading doses Long-term patients with stable disease	Take VA starting from smallest letter and work upwards to save time Pinhole vision may not be necessary
IOP measurement	Monitor glaucoma risk in IVT patients	Increased contact time with patient and staff Aerosolized droplets from non-contact/pneumatic tonometry	Undetected IOP rise	High risk glaucoma patients Cupped disc Post intravitreal steroid injection for the first time	Routine follow up No history of glaucoma or disc cupping Already has separate glaucoma follow-up appointment	Suspend the use of non-contact tonometry, use Goldmann applanation or I-care tonometry
Pupil dilation	Allows the examination of the peripheral retina	Increased contact time with patient and staff; spread of COVID-19 from contaminated eye drops	Risk of missing retinal pathology	Treatment naive High myopia Extra-foveal disease Visual field loss	Long-term patients with stable disease	Dilation eye drops should be administered only once on arrival, if needed patient can be given disposable minims of eye drops for repeated administration
OCT	Objective structural assessment of active disease Can be used to determine T&E decisions	Increased contact time with staff	Undetected Worsening disease activity Early recurrence with no VA loss not detected Missed screening of fellow eye	Treatment naïve 4 weeks after 3^rd^ loading dose	Patients receiving loading doses Long-term patients with stable disease Known maximum treatment interval	Plastic shield in machines where patient faces the technician Keep scanning protocol to a minimum Decentralise imaging service
Slit-lamp examination	Detection on non-retinal pathology Assessment of the retinal periphery Detection of new areas of bleeding	Increased close contact with staff	Undetected Non-retinal pathology and peripheral retinal pathology Undetected new retinal hemorrhages or rubeosis	Treatment naïve cases Patients with worsening visual acuity	Patients receiving loading doses Long-term patients with stable disease	Plastic shield in machines where patient faces the doctor N95 masks and goggles for high risk patients
Ophthalmology consultation	Direct reporting of symptoms Patient doctor rapport	Increased prolonged close contact with doctor	Undetected pathology not picked up by imaging	Treatment naïve cases	Patients receiving loading doses Long-term patients with stable disease	To be replaced by telephone or video consultation

VA: visual acuity, IOP: intra-ocular pressure, OCT: optical coherence tomography, loading doses refer to intravitreal anti-VEGF therapy

### Neovascular age-related macular degeneration

Multiple lines of evidence recommend that patients with nAMD in their first two years of treatment should be prioritised [5, 7]. Previous studies on the natural history of nAMD show that delaying IVT treatment results in vision loss(control arm of MARINA and ANCHOR) [13] and quarterly IVT after the 3 monthly loading doses anti-VEGF loading doses has inferior visual outcomes compared to monthly treatment (PIER and EXCITE study). Hence, an intensive treatment regimen for nAMD should be recommended from a vision standpoint in the treatment and consent discussions during the COVID-19 pandemic, despite the risk of being infected. For treatment naïve patients, OCT and/or OCT angiography (OCTA) should be preferred for confirming the diagnosis in place of dye angiography, which is time-consuming and requires increased person-to-person contact [7]. In nAMD patients, a modified T&E approach mixed with fixed dosing interval has been proposed to minimize the need for VA, OCT, and slit-lamp examination at every visit, while avoiding the risk of under-treatment [3]. An example of this was the TriPla regimen was proposed, which was a hybrid of fixed dosing and T&E, with an aim to still provide an individualized approach but minimizing the number of examinations and risk of COVID-19 exposure [15]. The ALTAIR study showed that increasing intervals by 4 weeks in a T&E regimen with aflibercept carried no differences in the visual outcomes or the number of injections compared to the traditionally adopted 2-week extension. Results from the FLUID study also showed that visual outcomes are comparable using a relaxed approach in patients versus a strict no tolerance to subretinal fluid approach. This meant that IVT interval for patients with stable VA and minimal stable subretinal fluid could continue to be extended as long as they did not deteriorate [14]. These added treatment strategies may also help in reducing the number of follow-up visits.

### Diabetic macular edema and diabetic retinopathy

Diabetic patients are at higher risk of COVID-19 complications; therefore, extra care should be provided to these patients to minimize the risk of infection. General recommendations for DME management are to defer all IVT treatments and follow-up unless the patient is monocular, has significant vision loss from DME, or has severe non-proliferative or proliferative diabetic retinopathy (in this case, pan-retinal photocoagulation should be considered) [5, 7]. Previous studies have shown that the long-term risk of vision loss in DME patients is lower than in nAMD (control/laser arms of RISE, RIDE, RESTORE, VIVID, VISTA) [16]. In treatment naïve patients, a delay in anti-VEGF IVT treatment may result in a higher risk of suboptimal long-term visual outcomes (crossover arms RISE, RIDE,VIVID, and VISTA, RESTORE extension study). Hence, for both treatment naïve and DME patient on treatment, guidelines state that follow-up appointments should be deferred, but should not be postponed for more than 4–6 months as this could lead to irreversible vision loss [7, 17]. When treatment is initiated, 6 monthly loading anti-VEGF injection doses (as recommended by the DRCRnet: Protocol T) can be performed as an injection-only appointment to reduce time spent in the clinic. Sustained-release intravitreal corticosteroid implants can also be considered as an alternative therapy in suitable patients to adequately treat DME and reduce the number of injections and follow-up visits, however additional visits for intra-ocular pressure checks may be required in higher risk cases.

### Macular edema related to retinal vein occlusion

Similar to DME, natural history studies show the risk of long-term vision loss from ME-RVO is low (control arms-VIBRANT, CRUISE, CORPENICUS, and GALILEO). Nevertheless, macular edema associated with central retinal vein occlusion (ME-CRVO) can be associated with a higher risk of suboptimal long-term visual outcomes in case of significant delay in anti-VEGF IVT treatment (crossover arms, CRUISE, COPERNICUS, and GALILEO). Recommendations for macular edema associated with branch retinal vein occlusion (ME-BRVO) is to defer all IVT treatments. Intensive monthly IVT anti-VEGF loading doses (done as injection only appointments) are recommended for the treatment naïve ME-CRVO [7]. In patients with ME-CRVO treated with monthly bevacizumab or ranibizumab, that have persistent activity or have recurrences, when monthly intervals are extended past 4 weeks, a switch to aflibercept or the dexamethasone implant may allow increased treatment intervals (NEWTON, SCORE 2).

### Reducing the risk of COVID-19 transmission within the IVT clinic

**Fig. 2:**
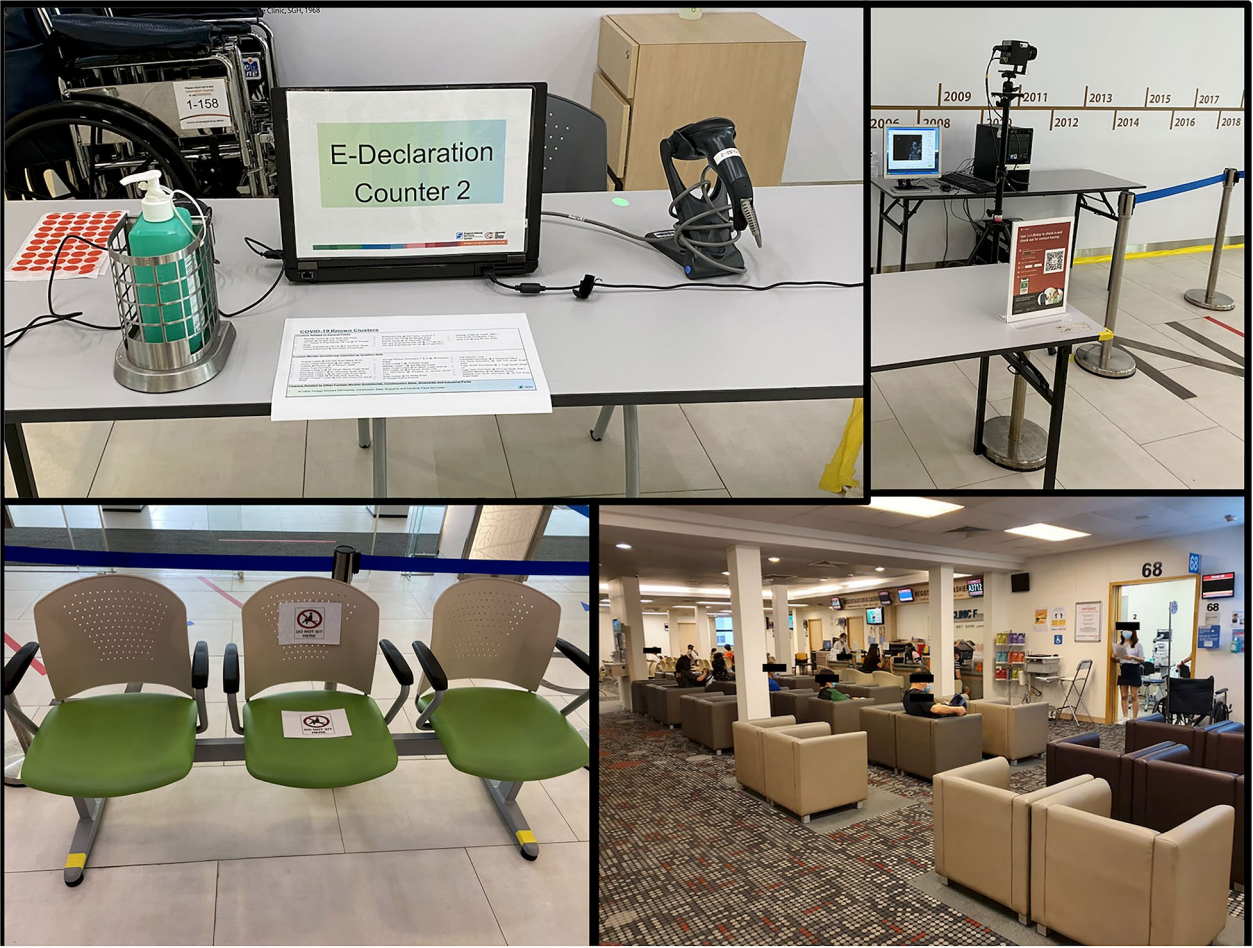
An example of a pre-screening counter for COVID-19 located at the entrance of the tertiary center (top left image), a government supported digital application (top right image) is used to record the patients entry details, symptoms, previous exposure to Covid-19 and travel history (also used for contact tracing if needed), an automatic thermal scanner (top right image) to detect patients with a fever as they enter the center. Examples of signs on clinic waiting room seats used to encourage social distancing (left image) and an example of patients in the waiting room of the clinic (right image) and staff wearing surgical masks and practicing social distancing

Recommendations to reduce the risk of COVID-19 transmission within the clinic include well-organized efforts to reschedule appointments for non-urgent patients, by giving them clear advice to postpone their visits and to contact the hospital only if their condition deteriorates or they require a prescription for drug-refill [2, 7]. Increased manpower should be provided for walk-in or emergency services, to address a potential rise in patients whose appointments have been rescheduled. Clear communications on public health recommendations should be given to patients before they attend the clinic, which include limiting accompanying persons, social distancing, hand hygiene, and wearing masks at all times (Fig. 2) [2, 7]. As countries start to relax confinement measures, patients will need to be continually reminded of the importance of maintaining a high degree of vigilance and compliance to all the public health recommendations specified above while within hospitals and clinics.

**Fig. 3:**
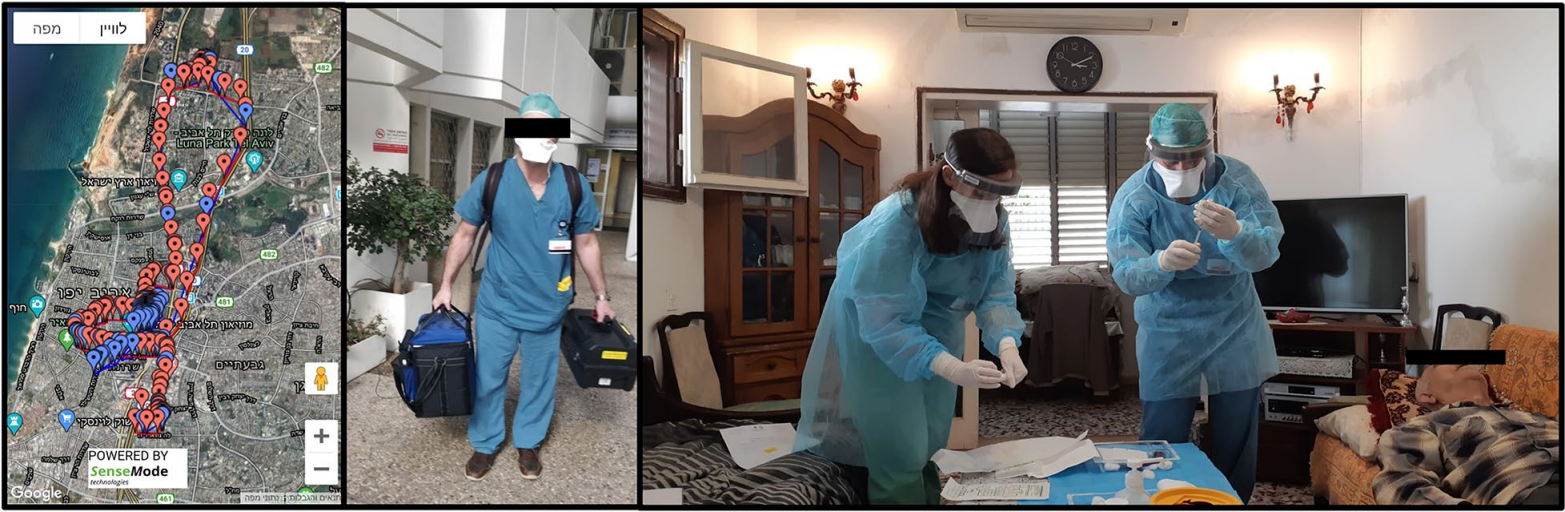
Decentralized home intravitreal therapy (IVT) service shown by the map illustrating the planned route of the home IVT service (left image), medical staff carrying the portable home IVT equipment (middle image) and medical team administering IVT to a patient at his home (left image

PPE is extremely important to prevent COVID-19 transmission, and it is recommended that at the very least surgical masks are worn by staff, patients, and caregivers [2, 7] (Fig. 3). Routine screening for respiratory symptoms, travels, or previous COVID-19 contact history, 2–14 days prior to the clinic visit, and temperature checks on arrival, have been recommended for all patients and caregivers before entering the eye clinic (Fig. 2) [2]. Some studies have suggested an increased risk of endophthalmitis associated with surgical masks worn by patients [18, 19], however a large multi-center study, showed no difference in the culture positive endophthalmitis rates between cohorts with no masks and those where both patients and physicians wore masks [20]. Prolonged mask wear of more than 4 h was also suggested as having a higher bacterial load that can be reduced with povidone iodine administration [21]. Taping of the top of masks or using a sealed drape before IVT administration has also been suggested as another alternative to decrease aerosolized particles from the patient’s mouth that may carry oral pathogens [22].

In general, all assessment procedures should be kept to a minimum and deferred where possible (Table 4). Suggested modifications to respective procedures are outlined to minimize the total time taken and contact with staff which include adequate social distance, with a clear outlined delineation between surfaces in contact with the patient and staff. A recent study described the development of a new intravitreal injection center based on “LEAN” principles (creating more value for customers with fewer resources, minimizing waste), resulting in better quality and efficiency, speed of the overall procedures and clinical capacity of the IVT service, with an aim to reduce the risk of COVID-19 transmission [23]. Recently revised recommendations released by the Vision Academy Steering Committee, included guidelines on IVT treatment based on the local epidemic pressure, to ensure the safety of patients and staff and the sustainability of healthcare resources, in era of easing COVID-19 measures leading to a resurgence of disease in many areas [17]. Apart from local epidemic pressure, the vaccination rates should also be considered when assessing the risk of COVID-19 transmission during IVT [17]. In particular, due to the long lasting effect of endemic COVID-19, there was an emphasis on maintaining treatment intervals wherever possible to avoid risk of permanent visual changes especially in patients with DME and BRVO who have had their treatment postponed for more than 6 months during the initial wave of the COVID-19 pandemic [17].

There also should be a shift towards telemedicine, with models of care such as virtual clinics, where clinical decisions are based on imaging such as color fundus photography and OCT. Patients are then contacted remotely and their management plan conveyed through phone, messaging service, or video consultation [17, 24–26]. In further efforts to reduce crowding in the tertiary centers, decentralization of services into the community, such as primary eye care centers, imaging centers, satellite clinics, and even home intravitreal services can be considered (Fig. 3) [24, 27]. The COVID-19 pandemic presents a unique opportunity to incentivize governments and insurance companies to provide healthcare remuneration for new services and initiatives [24].

### The effect of COVID-19 on IVT adherence rates and visual acuity outcomes of patients receiving IVT

The added challenges during the COVID-19 pandemic, such as the fear of visiting hospitals for appointments, difficulties with accessing healthcare, rescheduling missed appointments, and the reduced patient capacity of hospitals and eye clinics to maintain adequate social distancing may increase the risk of non-adherence to IVT. Overall, numerous studies worldwide report the adherence rates for IVT being reduced significantly during the COVID-19 pandemic [28–31]. One Italian study reported better adherence rates associated with younger patients, worser vision in fellow eye and during period of no lockdown [28]. A German study reported that during the first wave [32]of the pandemic, risk factors for poor adherence included low VA of the treated eye, high VA of the untreated eye, COVID-19 in the family and DME [31].

A French study also reported, that during lockdown, there was a relatively marked decrease in IVT procedures that did not return to pre-lockdown levels despite subsequent opening up [30]. Even though overall IVT numbers have decreased during the pandemic and immediately post-pandemic, it will be inevitable in the endemic COVID-19 era, that there will be a “rebound” number of patients who will need IVT treatment, that may have a delayed presentation with more advanced disease [33].

Fight Retinal Blindness Registry is a large international data base that published data from 8 countries showed that 6 month drop-out rates were higher for ME-RVO (28%), DME (27%) and lower for AMD (20%) [34]. Eyes with AMD loss more vision in proportion with the number of injections than eyes with DME or ME-RVO [34]. Other studies have also reported significant short term and long term vision loss in all patients receiving IVT [35], especially AMD patients who have had lapses in treatment due to COVID-19 [29, 32, 36]. Interestingly, one study reported that AMD eyes with active disease, with a high injection demand (intervals less than 6 weeks) were able to be extended to 10–12 weeks with stable VA, however when intervals were extended to more than 12 weeks there was a risk short term vision loss [37]. A Chinese study, reported that patients on the T&E regiment versus those on pro nata (prn) regimen showed better visual outcomes when their therapy was halted during COVID-19 especially in eyes with Type 1 neovascularisation [38]. Devastating VA outcomes due to submacular hemorrhages in AMD eyes have also been reported when IVT treatment was delayed due to COVID-19 [39, 40].

In contrast to the current UK guidelines, recommending delaying all ME-RVO injections [5], one UK-based study showed that when IVT was delayed and then restarted, more DME eyes were able to regain vision, however VA in nAMD and ME-RVO eyes were less likely to return to baseline [41]. Another study examining the short- and long-term effects of delayed IVT of more than 8 weeks, showed that in the short-term vision loss was more marked in the DR and CRVO eyes compared to nAMD, while long-term vision loss was more commonly observed in CRVO and nAMD eyes, with BRVO patients least effected by the IVT delay [42].

Patient adherence in this setting may be improved through other solutions that include digital interactive education programs, digital home monitoring programs [43], a hotline that gives direct access to doctors or nurses counselors, an online appointment scheduling service and private video consultation services [7].

## Conclusion

In this review, we summarize the current IVT recommendations during the COVID-19 pandemic and justify these recommendations based on previous published pivotal trials and current published studies, outlining the effects of the COVID pandemic on various retinal diseases treated with IVT. We describe the effect COVID-19 with both published reports and real-world examples from various tertiary centers around the world and suggest recommendations that may improve future resilience in providing continued IVT for patients with chronic retinal diseases despite challenges from the pandemic.

## Data Availability

Not Applicable.

## Abbreviations

IVT: Intravitreal injection therapy
WHO: World Health Organization
NAMD: Neovascular age-related macular degeneration
DME: Diabetic macula edema
ME-RVO: Macular edema associated with retinal vascular occlusion
VA: Visual acuity
OCT: Optical coherence tomography
OT: Operating theatre
Anti-VEGF: Anti-vascular endothelial growth factor
PPE: Personal protective equipment
T&E: Treat-and-extend
